# Alterations of Body Composition, Blood Morphology and Fibrinogen Concentration after Vibration Therapy in Older Adult Women: A Randomized Controlled Trial

**DOI:** 10.3390/jcm12206620

**Published:** 2023-10-19

**Authors:** Anna Kabata-Piżuch, Agnieszka Suder, Paulina Handzlik-Waszkiewicz, Aneta Teległów, Anna Marchewka

**Affiliations:** 1 Department of Anatomy, Faculty of Physical Rehabilitation, University of Physical Education, 31-571 Krakow, Poland; 2 Department of Health Promotion, Faculty of Physical Rehabilitation, University of Physical Education in Krakow, 31-571 Krakow, Poland; 3 Department of Clinical Rehabilitation, Faculty of Physical Rehabilitation, University of Physical Education, 31-571 Krakow, Poland

**Keywords:** body composition, blood parameters, fibrinogen, physiotherapy

## Abstract

Vibrotherapy is one of the methods of physical therapy. Vibration, like various forms of physical activity, affects metabolic processes and health. The aim of this study was to assess the influence of thirty vibration sessions on body composition, hematologic and rheological indexes of blood, and protein and fibrinogen concentration in elderly women’s blood. The study included 69 women, aged 60–70 years (mean age 64.6 ± 2.9), who were randomly and parallel assigned into: the vibrotherapy group 1 (G1) that took part in vibrotherapy on the Knees module, the vibrotherapy group 2 (G2) that took part in vibrotherapy on the Metabolism module, and the control group (CG) without interventions. In all patients, the following assessments were performed twice—baseline and after thirty vibrotherapy sessions: an assessment of body composition, a complete blood count with a hematology analyzer and erythrocyte aggregation by a laser-optical rotational red cell analyzer; total plasma protein and fibrinogen concentrations were established, respectively, by biuret and spectrophotometric methods. Intergroup (between groups) and intragroup (within each group) changes were statistically evaluated. After applying thirty vibration sessions, a decrease in body composition parameters (BM, body mass G1, *p* < 0.05; G2, *p* < 0.001 and FFM, fat free mass G1, *p* < 0.05; G2, *p* < 0.05) was confirmed in both intervention groups and BMI, body mass index in G2 (*p* < 0.05). It was found that, in G2, changes in erythrocyte aggregation indexes (T ½, half time kinetics of aggregation, *p* < 0.05 and AI, aggregation index, *p* < 0.05) and decrease of fibrinogen concentration (*p* < 0.05) took place. A series of thirty vibration sessions did not cause significant alterations in blood morphological parameters; therefore, vibrotherapy did not disturb hematological balance. Vibration sessions had a positive effect on BM, BMI, AG and fibrinogen concentration in the studied women, indicating the usefulness of this form of activation in older adults. Due to a decrease in FFM observed in the study, vibrotherapy should be employed in conjunction with physical exercise and other forms of physical activity in the group of older adults.

## 1. Introduction

Vibrotherapy is one of the methods of physical therapy. The stimulus used in it is periodic vibrations, i.e., a mechanical stimulus in the form of vibrations. It may involve the whole body of the patient (WBV) or only a selected part (LV). Vibration reflexively causes muscle contraction by stimulating α-motoneurons innervating muscle spindles, a phenomenon called the Tonic Vibration Reflex (TVR) [1,2]. The effect of vibration is to induce short, rapid changes in the length of the muscle-tendon complex; the reactions of muscles and tendons include a cyclic transition between eccentric and concentric contractions [3]. During the reflex, increased activation of α-motoneurons through the muscle spindle system, changes in corticospinal excitability and intracortical processes are recorded [4]. A vibratory stimulus can, therefore, like various forms of physical activity, affect metabolic processes and health [5,6,7]. In the study of Yang et al. [8], the impact of 24 sessions of vibration treatments, applied for 8 weeks, was assessed on body balance, functional mobility, muscle strength and power, bone density, range of motion of lower limb joints and fear of falling, and improvement was observed in all tested parameters. In turn, Machado et al. [9] conducted research on a group of women participating in vibration training for a period of 10 weeks and confirmed the beneficial effect of the treatments on increasing muscle mass in the examined elderly women.

Another important mechanism triggered by vibrotherapy is the influence on blood vessels. The analysis of literature done by Games et al. [10] indicates an increase of peripheral blood flow as the WBV effect, which improves oxygenation and nutrition of tissues. One of the mechanisms giving rise to the effect is vasodilation caused by the increased effect of nitric oxide on the vascular endothelium, leading to the relaxation of smooth muscles [11]. The results indicate that the application of vibration elicited a tendency for an increase in peripheral blood flow that was approximately 14% higher than that shown in the placebo condition [12]. Robbins et al. [13] points to a high level of sensitivity of the peripheral vascular system to vibration exposure.

In addition, vibration, by reducing platelet aggregation, prevents the formation of clots and supports the removal of cholesterol deposits in the vessels [14]. Cochrane et al. [15] showed a positive effect of vibration treatments, both in young and elderly people, on increasing the efficiency of the circulatory system, related to the ability to absorb oxygen. Vibration treatments have a beneficial effect on the metabolism comparable to physical activity of moderate intensity. The vibration also has been a suitable and efficient strategy with reduced cost for the management of several unhealthy age-related conditions [16,17]. Treatments are applied in diseases with metabolic problems, such as type 2 diabetes and metabolic syndrome, as well as in the post-menopausal period or in patients from intensive care units who are exposed to prolonged lying down [18,19].

The vibration is conducted through the ascending nerve pathways to the posterior horns of the spinal cord [20]. The effect of blocking the simultaneously flowing signals of pressure and touch in the posterior horns of the spinal cord is the basic mechanism of the analgesic effect of vibrations [21]. In addition, the vibration procedure affects the nervous system by decreasing mental tension and lowering blood pressure, and leads to normalization and deepening of breathing [22]. Vibration positively influences insomnia and sleep quality, so that elderly people more willingly participate in the activities of everyday life [23].

A significant obstacle to the comparison of the results obtained by different scientists is the methodology of the conducted research. An analysis of the literature indicates a necessity for precise selection of vibration parameters, i.e., frequency, intensity and amplitude of vibrations. Recently, a group of specialists compiled guidelines related to the use of vibration [24,25].

Biological changes occurring in late adulthood are retrograde. Their basis is the loss of reproductive capacity of cells and their gradual degeneration. The process of progressive disability of the aging body is mainly influenced by systemic changes, the clinical consequences of which intensify the disease states occurring in old age. Elderly individuals commonly experience detrimental consequences from prolonged bedrest confinement, including deconditioning and increased susceptibility to orthostatic intolerance [26]. Vibration therapy, when administered in a supine position, appears to represent an effective intervention for mitigating these adverse effects and potentially preventing orthostatic intolerance. The topic of this paper seems to be important due to the potential health benefits associated with the use of a modern method, i.e., vibration treatments. Considering the growing interest in, and dissemination of, these procedures, it is worth investigating further aspects related to the impact of vibration on the human body, as scientific information on its impact on blood hemorheological indices is not common.

The aim of this prospective, randomized and controlled study was to assess the influence of thirty vibrotherapy sessions on body composition, morphological and rheological blood parameters, the level of basic proteins and fibrinogen concentration in the blood of women aged 60–70 years. We hypothesize that vibration therapy, similarly to other forms of physical activity, affects metabolic processes and condition and is associated with improvement in body composition, blood count and fibrinogen concentration in the group of older adult women.

## 2. Materials and Methods

### 2.1. Sample

The study employed a rigorous scientific methodology by adopting a prospective, randomized and controlled trial design. A detailed description of the research methods was presented in a previous paper [27]. In this study, the primary objective was to assess the effects of cycloidal vibration therapy on elderly women. The study aimed to evaluate the alterations in various parameters, including body composition, hematological and rheological profiles, protein blood markers and fibrinogen concentration.

The research project received ethical approval from the Ethics Committee of the Regional Medical Chamber in Krakow (Approval number: 3/KBL/OIL/2019) and followed CONSORT guidelines. Furthermore, this study has been registered as a clinical trial in the Australian New Zealand Clinical Trials Registry (ANZCTR) under the registration number 12622000255785.

The study included 69 women, aged 60–70 years (mean age 64.6 ± 2.9) randomly assigned to the following groups:

- the vibrotherapy group 1 (G1), *n* = 23, participating in vibrotherapy on the Knees module,
- the vibrotherapy group 2 (G2), *n* = 23, taking part in vibrotherapy on the Metabolism module,
- the control group (CG), *n* = 23.

The experimental group of patients underwent cycloidal vibration therapy treatments, while the control group received no interventions.

In addition to being female and aged from 60 to 70, the subjects had to have no contraindications to physical activity (medical history), and all subjects provided written consent to participate in the study.

The exclusion criteria for this study included lack of consent to participate in examinations; the presence of motion limitations that rendered vibrotherapy impractical; advanced illnesses of blood vessels (aneurysm, thrombosis, atherosclerosis); conditions of recent heart attacks and strokes; conditions after bone fractures till complete adhesion; conditions after breaking tendons, ligaments, and muscles till fully regenerated; other treatments (endoprosthesis, implantation, reconstruction, and other surgeries) till completely healed; acute infections caused by bacteria, fungi, or viruses, including skin infections; abscesses; non-controlled hypertension; advanced renal calculi and gallstones; acute condition of multiple sclerosis; epilepsy; illnesses with vertigo; insufficient mental fitness; syringomyelia; hemorrhage; increased body temperature; or active cancer [27]. These criteria were used to ensure the safety and appropriate inclusion of participants in the study.

The recruitment process involved utilizing an invitation-based approach, specifically targeting older individuals (age from 60 to 70) who were attending routine visits to a general practitioner. The physician officially approved these individuals for participation in vibration therapy sessions. Group assignment was based on simple randomization after drawing sealed opaque envelopes from a container (assignment concealment procedures). The assignment to the intervention on the Knees and Metabolism modules was parallel. The implementation was conducted by a physiotherapist. These two types of modules were selected to target areas of the body often affected by medical conditions in older individuals. The subjects were recruited and underwent intervention from February 2019 to July 2019, ensuring that the study groups achieved the minimum required sample size. Due to the inherent nature of the intervention, blinding of the trial was not feasible. However, to minimize potential biases, the laboratory staff, the biostatistician and the team assessing outcomes were kept unaware of the group assignments.

The female participants were instructed not to engage in additional physical activities beyond their normal routine, and to refrain from altering their dietary habits or medication regimens throughout the duration of the experiment. These aspects were assessed in a survey questionnaire completed by the women. A comprehensive explanation regarding the study’s objectives and procedures was provided to the participants, along with the option to withdraw from the study at any stage without the need for justification. After the intervention period, 11 women did not show up for the scheduled follow-up visit.

**Figure 1:**
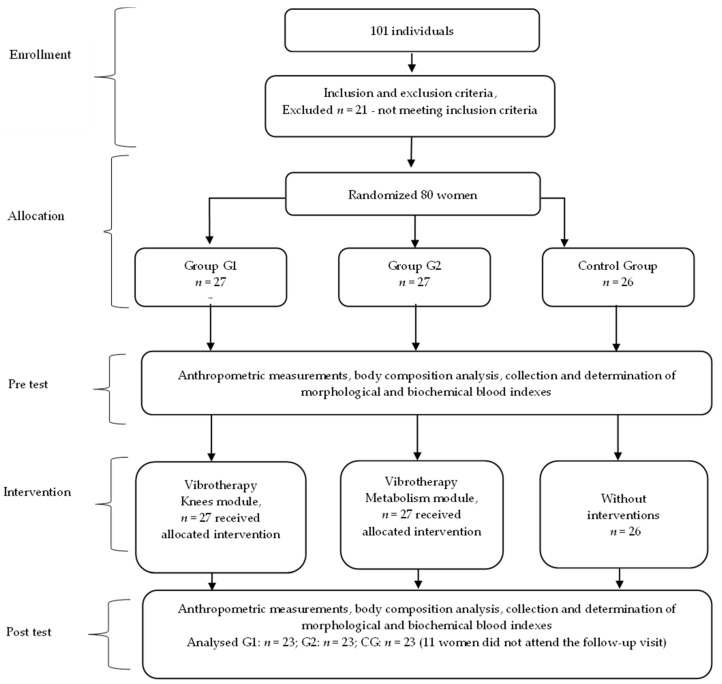
The flowchart of the study.

There were no occurrences of harm and adverse event reported during the trial period, and no potential side effects (headache, dizziness, nausea) were detected during the vibration treatment. The flowchart of the study is presented in Figure 1.

### 2.2. Methods

The primary outcome measures used in this study were an assessment of body composition, a complete blood count and fibrinogen concentration determination, and secondary outcome measures were an assessment of erythrocyte aggregation and total plasma protein. A comprehensive description of the research methods can be found in a previous publication [27]. In brief, all patients underwent a series of assessments that were conducted twice:

- Anthropometric measurements were directly obtained, including body height (Ht) in centimeters [cm] and body mass (BM) in kilograms [kg]. Height measurements were taken in a standing position, without shoes, with the head aligned in the Frankfurt plane using an anthropometer with a precision of 1 mm. Body mass was measured on a standardized medical scale, with an accuracy of 100 g, in a standing position. Body mass index (BMI) [kg/m^2^] was calculated according to the formula: BMI = body mass [kg]/height^2^ [m^2^].
- Body composition analysis was performed using the bioimpedance method with a Tanita analyzer (model Tanita BC-601, Tanita Corporation, Tokyo, Japan). The following parameters were assessed according to the manufacturer’s guidelines: fat free mass (FFM) [kg], body fat mass (BF) [kg], body fat percentage (BF) [%] and total body water (TBW) [%].
- The blood was collected before and 48 h after the last treatment, by a qualified laboratory diagnostician, under the supervision of a physician, in accordance with the applicable standards of the Laboratory of Blood Physiology of the University of Physical Education in Krakow. Fasting blood samples were collected in the morning from the basilic, cephalic or median cubital vein into EDTA tubes for whole blood hematology analysis; potassium edetate K_2_ (6 mL) was used as an anticoagulant, and a clotting activator was used for testing plasma; the main activator component was silicon dioxide SiO_2_ (6 mL). The material analyses were performed in the Laboratory of Blood Physiology of the University of Physical Education in Krakow, at the Department of Analytical and Clinical Biochemistry of the Oncology Institute in Krakow and in the Analytical Laboratory in Skawina.
- Using the ABX Micros 60 hematology analyzer (HORIBA, Sunnyvale, CA, USA), hematological blood parameters were assessed: red blood cell, white blood cell, platelet and reticulocyte parameters.
- A laser-optical rotary red cell analyzer (LORRCA) (R&R Mechatronics, Hoorn, The Netherlands) was used to study erythrocyte aggregation, and the results are presented as aggregation indices. The tests on the device were performed within 30 min of blood collection, at 37 °C, according to the standard protocol [28]. Parameters determining the kinetics of erythrocyte aggregation were examined: aggregation index (AI) [%], total degree of aggregation (AMP, amplitude) [IU], half-time kinetics of aggregation (T ½) [s].
- The concentration of fibrinogen (Fib) was determined using the Bio-Ksel Chrom-7 (Bio-Ksel, Grudziądz, Poland) apparatus through a spectrophotometric method. This involved the kinetic analysis of optical density changes during the coagulation reaction. The measurements were performed in grams per liter [g/L].
- Total protein [g/L] levels were estimated using the Roche Cobas 6000 analyzer (Roche, Basel, Switzerland) and the Proteinogram-Minicap Sebia analyzer (Sebia, Lisses, France), utilizing the biuret method. Plasma samples obtained from the collected blood were used to determine various protein fractions, including total proteins, albumins, α-1-globulins, α-2-globulins, β-1-globulins, β-2-globulins and γ-globulins.

### 2.3. Description of the Intervention

During the intervention, the participants in both groups received vibrotherapy sessions using mattresses (Vitberg, Nowy Sacz, Poland) that generated oscillatory-cycloid vibrations. The device has a TUV Rheinland certificate (No. 0197) and a quality certificate for class IIa medical devices (No. HD60118119001).

The RAM Vitberg+ system employed a combination of whole-body vibrations (WBV) using the Vitberg+ Base Module, two-segment, covering the area of the torso, upper limbs and thighs, with an additional local effect: the RAM Vitberg+ Knees therapeutic module, aimed at the knee joint area, as well as the Vitberg+ Metabolism RAM module. During operation, this module is characterized by the highest frequency of work in relation to the epigastrium, the middle part of the abdomen, the pubic, inguinal and subcostal areas and the subcutaneous fascia. These modules delivered localized vibration stimuli to these specific areas to produce therapeutic effects.

In G1, subjected to vibration using the Knees module, vibrotherapy was carried out in a semi-reclining position using the RAM Vitberg+ Base module together with the RAM Vitberg+ Knees module (Vitberg, Nowy Sacz, Poland), which is classified as an active class I medical device [29].

In G2, procedures were performed in the prone position with the use of the Vitberg+ Base Module reinforced with the Vitberg+ Metabolism RAM module (class I active medical device), (Vitberg, Nowy Sacz, Poland) [29].

**Figure 2:**
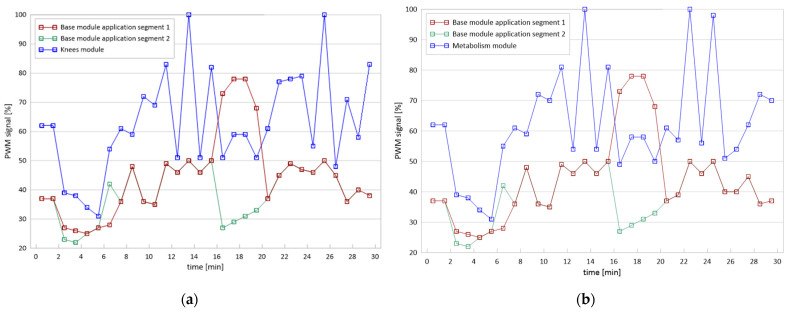
Voltage-current characteristics (PWM: Pulse Width Modulation) (**a**) of the Knees module and (**b**) of the Metabolism module; 100% fill indicates the maximum operating frequency of the module in given conditions.

The therapeutic stimulus was delivered through cycloid vibrations generated in three perpendicular directions (3D), resulting in intermittent pulsations with variable values of frequency [f], amplitude (A) and acceleration [a], which ranged from 10.10 to 52.20 Hz, 0.1–0.5 mm and 6.9–13.5 m/s^2^, respectively. The rates of applied vibration were time-variable according to the characteristics programmed by the manufacturer (Figure 2).

**Figure 3:**
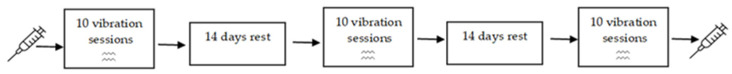
The vibrotherapy sessions.

The vibrotherapy protocol consisted of three courses, with each course comprising ten vibration sessions followed by fourteen days of rest. Vibrotherapy sessions were conducted five times a week, once per day. Each vibration treatment lasted 29 min and involved the implementation of eight microprograms (Figure 3).

All sessions were administered consistently in a controlled environment within the physiotherapy office in Skawina. The sessions were conducted by the same experienced physiotherapist, ensuring consistency and minimizing inter-operator variability. The therapy sessions took place in a dedicated room with consistent temperature (24 °C) and humidity (50%) levels. Furthermore, all sessions were scheduled at the same time of day, specifically in the morning, to minimize any potential diurnal variations that may influence the outcomes.

### 2.4. Statistical Analysis

The distribution of results for the analyzed variables was checked by applying a Shapiro–Wilk test. For variables with a normal distribution, the homogeneity of variance was also assessed using Levene’s test. Differences between the study groups and the control group were assessed using the F-test of the analysis of variance (ANOVA) or, if the assumptions were not met, the Kruskal–Wallis test. The least significant difference (LSD) test was used to evaluate multiple (post hoc) comparisons. Dependent variables were compared with Student’s *t*-test for related variables, and—in the case of failure to meet the assumptions—with the Wilcoxon test.

The effect size (ES) was calculated using the ηp^2^ coefficient, which represents the ratio of the sum of squares for the effect to the sum of squares for the error. The interpretation of the ES was as follows: 0.01 ≤ 0.05 (low effect), 0.06 ≤ 0.13 (moderate effect) and ≥0.14 (high effect). Meanwhile, for parameters with heterogeneous variances, the ES was calculated as the ratio of the Wilcoxon test statistic to the square root of the sample size [30].

The intraclass correlation coefficient (ICC) was calculated according to absolute agreement with two-way random effects model with interpretation: below 0.4—poor compatibility, 0.4–0.6—average compatibility, 0.6–0.75—high compatibility, 0.75–1—very high compatibility [31].

The number of participants required to demonstrate statistical significance was based on previously published studies in the field. The probability of error (α) of 0.05, the power (1 − β) of 0.80 and the mean effect size (d) of 0.8 were used to calculate the sample size. The analysis was carried out according to the originally assigned groups. The statistical significance of differences was assumed for a level of *p* < 0.05. The STATISTICA 13 package (StatSoft, Inc., Tulsa, OK, USA) was used for calculations.

## 3. Results

The study included a total of 69 women distributed into three groups. Group G1 consisted of 23 women with a mean age of 64.00 ± 3.13 years, who received thirty vibrotherapy sessions utilizing the Knees module over a period of thirty vibration sessions. Group G2 consisted of 23 women with a mean age of 65.3 ± 2.65 years, who underwent thirty vibrotherapy sessions utilizing the Metabolism module over the intervention period. The control group (CG) consisted of 23 women with a mean age of 64.57 ± 2.95 years, who did not receive any intervention (Table 1). Prior to the interventions, no significant differences were observed between the control group (CG) and the examined groups (G1 and G2) in terms of age, height and body composition. Additionally, no significant differences were found in initial parameters such as blood morphology, erythrocyte aggregation indexes, proteinogram (excluding total proteins) and fibrinogen levels between CG and the examined groups (G1, G2) (Table 1, Table 2, Table 3, Table 4, Table 5, Table 6 and Table 7). No adverse events were reported during and after the intervention.

Following the completion of three courses of vibrotherapy, significant reductions were observed in specific body composition parameters in G1, including BM (*p* = 0.006) and FFM (*p* = 0.013). In G2, significant decreases were observed in BM (*p* < 0.001), BMI (*p* = 0.017) and FFM (*p* = 0.011), confirmed by high or moderate effect size (ES) between the first and second examinations. However, no significant changes in the analyzed parameters were observed in the control group (CG) after the observation period (Table 2).

After the applied interventions, no changes were found in the level of parameters of red blood cells; however, for RBC (*p* = 0.045) and MCV (*p* = 0.012), the parameter values decreased in the CG after the period of observation (Table 3).

In the white blood cell and platelet parameters, higher WBC values (*p* = 0.021) were confirmed in G1 after thirty vibrotherapy sessions compared with G2 (*p* = 0.020) and GC (*p* = 0.013). MPV values decreased (*p* = 0.007) in G2 after the applied intervention. In the CG, no significant changes in the analyzed parameters were found after thirty vibration sessions (Table 4).

In the reticulocyte parameters, a significant decrease in the HFR, MFR and IRF fractions was demonstrated, as well as an increase in the LRF fraction, for all analyzed groups after thirty vibration sessions. No changes in RETC were confirmed in any of the study groups, but the effect size (ES) was high (Table 5).

Following the completion of thirty vibrotherapy sessions, significant findings were observed in the examined groups. In group G2, there was a notable increase in the half-life time (T ½) of erythrocytes (*p* = 0.030), indicating a slower rate of erythrocyte aggregation. Additionally, the aggregation index (AI) showed a significant decrease (*p* = 0.009), confirmed by high effect size (ES, ηp^2^ = 0.151), indicating a reduction in erythrocyte aggregation propensity. Conversely, no significant changes in these analyzed parameters were observed in the control group (CG) after an almost three-month period (Table 6). Furthermore, it was found that after three courses of sessions, there was a significant decrease in fibrinogen levels (*p* = 0.031), confirmed by high effect size (ES, ηp^2^ = 0.194), among the women who received vibrotherapy on the Metabolism module (G2). A similar change also occurred in CG (*p* = 0.007) (Table 6).

After thirty vibration sessions, significant changes in protein levels were observed specifically in the case of total proteins. In group G2, a decrease in total protein values was found (*p* = 0.010), but it remained within the normal range. However, in the control group (CG), a decrease in total proteins (*p* < 0.001), β-2-globulins (*p* = 0.036) and γ-globulins (*p* = 0.042) was observed after the period of observation (Table 7).

## 4. Discussion

The aim of this study was to assess the influence of thirty vibration sessions on body composition, hematologic and rheological indexes of blood, and protein and fibrinogen concentration in older adult women’s blood. The treatments affected body composition and fibrinogen concentrations in the examined women. After applying thirty vibration sessions, a decrease in body composition parameters (BM and FFM) was confirmed in both intervention groups, and in BMI in group G2, which took part in vibrotherapy on the Metabolism module. A series of thirty vibration sessions did not cause significant alterations in blood morphological parameters. However, it was found that in G2, changes in erythrocyte aggregation indexes (T ½, AI) and a decrease of fibrinogen concentration took place.

In our research, a significant decrease in body mass was confirmed in women undergoing vibration treatments, as well as in the BMI in the group subjected to vibration sessions on the Metabolism module. Yoo et al. [32] studied the impact of vibration training performed three times a week for 3 months on a group of 91 people and did not confirm a significant impact of vibrotherapy on reducing body weight and fat mass, or on increase of muscle mass.

The results related to body mass or BMI obtained in our research should be supplemented with an analysis of changes in subsequent body composition parameters, which also showed a decrease in FFM in the groups subjected to vibrotherapy, which was associated with a decrease in body mass and BMI in the surveyed women. It should be noted, however, that changes in FFM over time were not confirmed by the interaction between the groups. A review of data from the literature shows a beneficial effect of the applied vibrations on the muscle mass of the subjects. The vibration activates the muscle spindles causing muscle contraction (tonic vibration reflex—TVR) [33]. Bogaerts et al. [34] conducted a study whose aim was to assess the effect of vibration training, lasting a year, on cardio-respiratory fitness and muscle strength in 220 people over the age of 60. Patients were assigned to two groups with intervention in the form of vibration sessions and fitness exercises, and the third was a control group, without intervention. An increase in muscle strength was demonstrated in both intervention groups. Mikhael et al. [35] reviewed the literature on the impact of different vibration frequency ranges on changes in muscle mass. The collected publications contained research using vibrations in the range of 12–50 Hz. The increase in muscle mass was confirmed using frequencies in the range of 26–28 Hz [36], whereas application of vibration frequencies of 30–50 Hz did not cause changes in muscle mass [37]. In our research, the frequency ranged from 10.10 Hz to 52.20 Hz, and the vibrotherapy was carried out in a sitting position or lying on the stomach, i.e., in unloading positions. Based on the direction of changes in the FFM values in the examined women and data from the literature, it seems reasonable to use vibration treatments in combination with regular physical exercise, especially in the group of elderly people.

Parameters such as whole blood and plasma viscosity, erythrocyte deformability and the ability of erythrocytes to aggregate are responsible for proper blood flow [38]. Compared with younger people, older people have a lower hematological value, which means a decrease in the number of blood cells, hemoglobin level and mean red blood cell volume (MCV). Coppola et al. [39], conducting research on a group of people aged 19–102, showed lower levels of hemoglobin, erythrocytes and platelets in people over 60 compared with younger people. In our own research, after a series of thirty vibration sessions, no changes in the mean values of red blood cell indices in the groups of women subjected to the intervention were confirmed. In the control group, however, there was a significant decrease in the number of red blood cells (RBC) and mean red blood cell volume (MCV) after the period of observation. In the studies of Theodorou et al. [40], conducted on a group of 28 healthy women using vibration training for 8 weeks at a frequency of 25 Hz, also no changes in the level of erythrocytes, leukocytes and platelets were confirmed. However, studies conducted on a group of physically active people showed an increase in the concentration of erythrocytes and hemoglobin compared to people leading a sedentary lifestyle [41]. Regular aerobic training lasting longer than 12 weeks caused a decrease in haemoglobin level [42]. The consistent engagement in physical training elicits a physiological response within the body characterized by a notable augmentation in plasma volume, often reaching up to a 20% expansion. This phenomenon is intricately linked with a concomitant reduction in hematocrit levels and a decrease in blood viscosity. Furthermore, the enduring consequences of sustained physical exercise encompass heightened erythrocyte deformability and a diminished propensity for erythrocyte aggregation. These alterations collectively contribute to the facilitation of smoother blood flow within the circulatory system [43].

After thirty vibration sessions, the group subjected to vibration on the Knees module was characterized by higher WBC values—the number of white blood cells in relation to the other groups—but in this group, at the beginning of the study, this parameter was elevated in relation to those of the second intervention group and the control group. It should also be noted that the WBC values still oscillated within the normal range in the study groups. In the research of Bobeuf et al. [44], the effect of aerobic training lasting 6 months on the white blood cell count in 29 people aged 61–73 was not confirmed. Physical exertion leads to an increase in the number of leukocytes in the circulating blood. This phenomenon arises from accelerated blood circulation, vasodilation and increased lymph flow, which results in the return of leukocytes attached to the walls of blood vessels, as well as those located in internal organs, into peripheral circulation. The release of adrenaline in response to physical exertion causes an immediate rise in leukocyte counts in the blood during the initial minutes of exertion, and the subsequent release of cortisol is responsible for further increases, particularly in neutrophils. Prolonged and recurrent moderate physical exertion leads to an adaptation of the leukocyte system to the applied stimulus [45].

The own results regarding the level of reticulocytes indicate that the applied vibration sessions do not stimulate hematopoietic activity of the bone marrow—in all analyzed groups, as well as in the control group, a decrease in the level of immature and medium-mature reticulocytes was noted, as well as an increase in the level of mature reticulocytes after intervention and observation. The relative number of reticulocytes did not change significantly in the analyzed groups. With the advancement of the aging process, there is a decrease in the volume of bone marrow used for hematopoiesis and a decrease in the proliferative activity of stem cells, which results in reduced production of erythrocytes [46]. The applied intervention in the form of vibration sessions did not stimulate erythropoiesis, whereas in physically active people, increased concentrations of immature forms of erythrocytes and stimulation of their production by the bone marrow were confirmed [47]. A moderate aerobic training lasting for 6 weeks was associated with an increased fraction of young erythrocytes [48].

In terms of blood aggregation indices in the group subjected to vibration treatments on the Metabolism module, an increase in the T_1/2_ parameter was confirmed in the study after thirty vibration sessions, i.e., an increase in the time necessary to achieve half of the maximum aggregation. A reduction in the aggregation index (AI) was also confirmed in the G2 group, while no significant changes were observed in the control group. A similar downward trend of AI was shown in the group of men aged 60–70, subjected to vibrotherapy on the Metabolism module in our previous research, but not confirmed statistically [27]. Aloulou et al. [49] conducted a study involving the use of 8-week aerobic training in a group of overweight elderly people. These studies showed no significant changes related to the formation of aggregates. A decrease in the aggregation index under the influence of a 6-week resistance training was observed by Cakir-Atabek et al. [50]. They also noticed a simultaneous increase in the AI under the influence of a single intensive training session.

Tripette et al. [51] noticed a relationship between aggregation and water loss by erythrocytes. Dehydration contributes to the thickening of the blood. Proper hydration of the body during exercise should be used to reduce blood viscosity because prolonged dehydration affects the destabilization and increases the stiffness of the erythrocyte cell membrane. In our own research, no significant differences between the analyzed groups in the percentage of plasma volume (PV)—calculated on the basis of hemoglobin concentrations and hematocrit numbers before and after a series of thirty vibration sessions and a three-month observation—were confirmed: in the CG, the mean growth in PV was 1.2%, whereas in G1 and G2 it was 0.4% and 0.73%, respectively (results not included).

The results of our research indicate a significant reduction in the level of the average fibrinogen concentration from 3.50 to 3.20 [g/L] in the group of examined women subjected to vibration sessions on the Metabolism module. Similar studies were carried out in a group of elderly men subjected to a series of thirty vibration sessions, also on the Metabolism module, and a decrease in fibrinogen was confirmed in the group with the vibration applied, while in the control group, an increase in fibrinogen concentration was shown during the period of observation [27]. However, relatively few studies show the influence of vibration treatments on fibrinogen concentration and changes in rheological parameters of the blood [10,52]. Ghazalian et al. [53], after 5 weeks of vibration treatments, demonstrated an indirect effect of vibration treatments on the level of fibrinogen concentration by enhancing endothelial function. Vibration generates pulsating shear forces on the endothelium, resulting in increased blood flow and increased endothelial nitric oxide synthase (eNOS) activity and nitric oxide (NO) concentrations [54]. The treatments cause a decrease in the level of erythrocyte aggregation and a decrease in the level of fibrinogen [55].

In response to the vibration treatments in the examined women, no changes were found in most protein indices, apart from a decrease in the level of total protein in the group of women subjected to vibration using the Metabolism module (Vitberg, Nowy Sacz, Poland). The total protein level in the serum of elderly people decreases gradually with age, and this process occurs faster in women than in men [56]. However, in our research, the level of total protein was in the range of 65.8–83.6 [g/L], i.e., it fluctuated all the time within the normal range of 60–80 [g/L] [57]. In the study of males aged 60–70 years, Kabata-Piżuch et al. [27] did not confirm the influence of vibration treatments on the Metabolism module of the same parameters on the change in protein indices. Yanagita et al. [58] described the risk of “brittle bones” in the elderly, which may be caused by low levels of proteins, mainly albumin. In our research, no significant changes in the level of albumin and globulin levels were observed in the intervention groups, while in the control group, a decrease in the fraction of gamma globulin, beta-2-globulin and total protein was confirmed after the period of observation. Therefore, the use of vibration treatments did not lead to pathophysiological changes, and the balance between the production and breakdown of the main protein fractions in these groups was maintained.

An undoubted limitation of the project was the lack of simulation of vibration treatments, related to the lack of placebo modules in the control group. There was also no blinding during randomization and patients were aware of the type of the applied intervention. Furthermore, the absence of control over the dietary intake adopted by participants may have constituted a confounding factor influencing the observed outcomes.

## 5. Conclusions

In conclusion, it should be emphasized that the vibrotherapy program positively influenced the mean concentration of fibrinogen in older adult women and resulted in lowering the body mass and BMI, confirming some of the hypotheses. Due to the decrease in FFM shown in the studies, vibrotherapy should be used in conjunction with physical exercises and other forms of physical activity in the group of older adults. A series of thirty vibration sessions did not cause any significant changes in the morphological parameters of the blood in the subjects, so vibrotherapy did not disturb the hematological balance of the blood. Reducing the aggregation index in the group subjected to vibrotherapy with the Metabolism module is a beneficial phenomenon, and additional investigations should be conducted to assess the efficacy of vibrotherapy, ideally in a multicenter, double-blind, randomized controlled trial, with the potential inclusion of a placebo device, and a more extensive patient cohort. Such endeavors would yield valuable insights into the translation and incorporation of vibrotherapy into routine healthcare practices.

## Figures and Tables

**Table 1:** Values of age and height in the examined (G1, *n* = 23; G2, *n* = 23), and control (CG, *n* = 23) groups before vibrotherapy series (pre), considering interactions between the groups (*p* int).

Variables	Group	Pre				
		$\bar{X}$	SD	Me	Min	Max
Age [years]	G1	64.04	3.13	64.00	60.00	70.00
	G2	65.30	2.65	66.00	60.00	69.00
	CG	64.57	2.95	64.00	60.00	70.00
	*p* int	0.321				
Height [cm]	G1	162.00	0.09	160.00	147.00	188.00
	G2	163.00	0.06	164.00	151.00	175.00
	CG	163.00	0.07	164.00	151.00	181.00
	*p* int	0.859				

$\bar{X}$ —mean, SD—standard deviation, Me—median, Min—minimum, Max—maximum, *p*—statistically significant value (*p* < 0.05).

**Table 2:** Values of body composition parameters in the examined (G1, *n* = 23; G2, *n* = 23), and control (CG, *n* = 23) groups before and after vibrotherapy series, considering interactions between the groups (*p* int) and changes over time (*p* pre-post).

Variable	Group	Pre						Post						ES	*p* Value
		$\bar{X}$	SD	Me	Min	Max	CV	$\bar{X}$	SD	Me	Min	Max	CV		Pre-Post
BM [kg]	G1	76.27	14.52	74.50	46.90	103.40	19.03	75.82	14.33	74.50	47.00	103.00	18.90	0.291	0.006 *
	G2	77.23	11.57	74.00	52.70	102.50	14.98	75.71	10.61	73.10	53.10	97.60	14.01	0.477	<0.001 *
	CG	77.00	12.25	77.10	55.10	98.80	15.91	76.78	12.10	76.60	55.20	97.10	15.76	0.038	0.379
	*p* int	0.965						0.950							
	ICC	−0.39													
BMI [kg/m^2^]	G1	29.03	5.43	28.92	19.03	38.92	18.71	29.19	5.67	29.00	19.07	38.30	19.43	0.011	0.628
	G2	29.60	5.55	28.84	22.51	46.68	18.75	28.39	4.05	27.97	22.68	37.19	14.28	0.231	0.017 *
	CG	29.19	4.65	29.40	20.49	35.9	15.94	29.10	4.55	29.28	20.52	35.34	15.64	0.042	0.345
	*p* int	0.931						0.825							
	ICC	−0.47													
FFM [kg]	G1	43.89	6.89	43.90	33.40	58.80	15.69	43.38	6.82	43.40	33.70	59.20	15.72	0.249	0.013 *
	G2	43.43	6.12	44.50	29.90	52.80	14.09	42.99	5.67	44.10	29.80	52.10	13.20	0.061	0.011 *
	CG	44.15	7.75	44.10	26.70	58.70	17.55	43.64	7.29	44.00	26.50	57.40	16.70	0.015	0.567
	*p* int	0.938						0.945							
	ICC	−0.27													
BF [kg]	G1	30.20	10.93	30.40	8.80	52.90	36.19	32.70	5.52	32.10	25.30	42.80	16.87	0.060	0.248
	G2	31.40	9.83	30.90	17.10	56.30	31.31	32.50	5.14	33.40	22.30	40.20	15.81	0.013	0.593
	CG	30.70	10.30	30.10	14.90	48.90	33.58	33.50	6.31	33.20	20.30	45.20	18.81	0.055	0.272
	*p* int	0.926						0.809							
	ICC	0.40													
BF [%]	G1	38.58	8.90	33.16	18.70	60.20	23.10	38.69	8.98	40.10	18.60	59.20	23.20	0.034	0.397
	G2	40.08	8.35	32.08	23.80	54.90	20.80	40.04	8.48	40.20	22.30	55.10	21.20	0.001	0.893
	CG	39.15	9.82	32.40	21.40	63.00	25.10	39.27	9.69	38.30	23.60	63.40	24.70	0.016	0.559
	*p* int	0.852						0.879							
	ICC	−0.04													
TBW [%]	G1	44.59	6.12	43.20	29.30	57.90	13.72	43.72	5.67	42.10	29.80	57.70	12.97	0.219	0.121
	G2	43.07	4.91	43.60	34.70	55.80	11.40	43.01	4.71	43.50	34.20	54.80	10.94	0.016	0.553
	CG	44.04	6.87	44.90	26.70	56.90	15.59	44.02	6.91	44.90	26.50	57.10	15.70	0.007	0.724
	*p* int	0.686						0.836							
	ICC	−0.45													

$\bar{X}$ —mean, SD—standard deviation, Me—median, Min—minimum, Max—maximum, CV—coefficient of variation, ES—effect size, ICC—intraclass correlation coefficient, *p*—* statistically significant value (*p* < 0.05), BM—Body Mass, BMI—Body Mass Index, FFM—Fat Free Mass, BF—Body Fat, TBW—Total Body Water.

**Table 3:** Values of red blood cell parameters the examined (G1, *n* = 23; G2, *n* = 23), and control (CG, *n* = 23) groups before and after vibrotherapy series, considering interactions between the groups (*p* int) and changes over time (*p* pre-post).

Variable	Group	Pre						Post						ES	*p* Value
		$\bar{X}$	SD	Me	Min	Max	CV	$\bar{X}$	SD	Me	Min	Max	CV		Pre-Post
HB [g/dL]	G1	13.87	0.88	13.70	12.10	15.50	6.33	13.96	0.87	14.00	11.70	15.20	6.25	0.041	0.341
	G2	13.90	1.75	13.90	7.70	16.50	12.61	13.86	1.03	14.10	11.80	15.50	7.41	0.001	0.884
	CG	13.60	0.90	13.30	12.10	15.70	6.64	13.46	0.79	13.50	11.90	15.20	5.91	0.089	0.158
	*p* int	0.666						0.142							
	ICC	0.60													
HCT [%]	G1	41.26	2.41	40.80	36.80	45.40	5.84	41.24	2.39	41.20	36.20	45.70	5.79	0.000	0.982
	G2	41.01	4.96	41.10	24.10	48.60	12.11	40.91	2.89	41.50	35.20	45.60	7.07	0.001	0.890
	CG	40.13	2.53	40.10	35.70	44.70	6.30	40.13	3.73	39.80	34.60	53.40	9.30	0.000	1.00
	*p* int	0.520						0.451							
	ICC	0.53													
RBC [T/L]	G1	4.61	0.65	4.46	3.96	7.30	14.02	4.53	0.36	4.51	3.90	5.60	7.85	0.009	0.653
	G2	4.52	0.44	4.49	3.52	5.42	9.77	4.52	0.34	4.55	3.97	5.17	7.43	0.000	0.976
	CG	4.65	1.14	4.36	3.95	9.70	24.59	4.51	0.61	4.38	3.95	6.90	13.60	0.043	0.045 *
	*p* int	0.869						0.987							
	ICC	−0.30													
MCV [fl]	G1	91.35	3.46	91.50	85.10	99.10	3.79	91.00	3.46	90.50	83.60	98.90	3.80	0.005	0.747
	G2	90.35	5.66	90.90	68.50	98.50	6.26	90.54	3.58	90.50	81.00	97.50	3.95	0.005	0.748
	CG	91.20	3.21	90.90	83.80	97.70	3.52	90.73	3.21	9.20	83.10	97.80	3.54	0.220	0.012 *
	*p* int	0.694						0.902							
	ICC	0.10													
MCH [pg]	G1	30.75	1.34	30.70	27.90	33.70	4.35	30.74	1.52	31.00	27.00	33.70	4.95	0.000	0.984
	G2	30.60	2.18	30.90	21.80	33.40	7.12	30.61	1.34	30.90	26.80	33.00	4.38	0.000	0.986
	CG	30.62	1.15	30.40	28.00	33.00	3.74	30.89	1.62	30.60	27.90	30.20	5.24	0.067	0.221
	*p* int	0.943						0.815							
	ICC	−0.24													
MCHC [g/dL]	G1	33.65	0.49	33.60	32.80	34.90	1.45	33.92	0.71	34.00	32.30	35.10	2.10	0.093	0.147
	G2	33.88	0.71	33.90	31.90	35.30	2.09	33.81	0.61	33.90	32.50	35.10	1.81	0.016	0.566
	CG	33.65	0.64	33.50	32.80	35.30	1.91	33.84	0.86	34.10	31.80	35.20	2.53	0.068	0.219
	*p* int	0.346						0.882							
	ICC	−0.06													
RDW-CV [%]	G1	13.90	0.50	13.90	12.80	15.10	3.61	14.00	0.57	13.80	13.10	15.70	4.10	0.006	0.712
	G2	13.80	0.71	13.70	12.60	15.70	5.18	14.30	2.44	13.80	12.90	25.20	17.08	0.062	0.242
	CG	13.60	0.48	13.50	12.80	14.60	3.49	13.60	0.56	13.70	12.50	14.60	4.13	0.002	0.841
	*p* int	0.254						0.332							
	ICC	0.56													
RDW-SD [fl]	G1	46.50	1.71	46.50	42.80	50.10	3.67	46.30	1.88	45.80	44.00	50.10	4.06	0.092	0.650
	G2	45.60	2.74	45.80	39.20	49.40	6.02	45.90	2.85	46.50	40.50	50.10	6.20	0.277	0.120
	CG	46.00	2.32	46.50	41.40	49.40	5.04	45.70	2.15	46.50	41.30	49.40	4.71	0.263	0.242
	*p* int	0.375						0.707							
	ICC	0.43													

$\bar{X}$ —mean, SD—standard deviation, Me—median, Min—minimum, Max—maximum, CV—coefficient of variation, ES—effect size, ICC— intraclass correlation coefficient, *p*—* statistically significant value (*p* < 0.05), HB—Hemoglobin, HCT—Hematocrit, RBC—Red Blood Cells, MCV—Mean Corpuscular Volume, MCH—Mean Corpuscular Hemoglobin, MCHC—Mean Corpuscular Hemoglobin Concentration, RDW-CV—Red Blood Cell Distribution Width, RDW-SD—Red Blood Cell Distribution Width-Standard Deviation.

**Table 4:** Values of white blood cells and platelet parameters in the examined (G1, *n* = 23; G2, *n* = 23), and control (CG, *n* = 23) groups before and after vibrotherapy series, considering interactions between the groups (*p* int) and changes over time (*p* pre-post).

Variable	Group	Pre						Post						ES	*p* Value
		$\bar{X}$	SD	Me	Min	Max	CV	$\bar{X}$	SD	Me	Min	Max	CV		Pre-Post
WBC [10^9^/L]	G1	6.00	1.23	5.70	4.10	8.30	20.53	6.30	1.27	6.30	4.20	8.70	20.18	0.031	0.410
	G2	5.36	1.15	5.30	3.60	8.20	21.45	5.47	1.07	5.50	3.60	7.80	19.59	0.012	0.606
	CG	5.50	1.30	5.00	3.60	8.10	23.72	5.41	1.20	5.10	3.90	7.80	22.16	0.009	0.655
	*p* int	0.182						0.021 *							
	ICC	0.84													
PLT [10^9^/L]	G1	244.3	89.15	234.0	127.0	513.0	36.49	266.7	107.20	238.0	146.0	664.0	40.19	0.027	0.444
	G2	239.1	69.47	234.0	152.0	375.0	25.29	251.3	66.08	238.0	132.0	380.0	26.30	0.103	0.126
	CG	243.6	57.82	235.0	145.0	363.0	23.74	251.6	64.89	242.0	114.0	400.0	25.79	0.154	0.058
	*p* int	0.964						0.765							
	ICC	−0.11													
MPV [fl]	G1	12.20	1.36	12.00	10.30	15.00	11.10	11.90	1.34	11.30	10.30	14.30	11.23	0.026	0.454
	G2	12.00	0.85	12.10	10.10	13.00	7.13	11.70	0.94	12.00	9.90	13.20	8.01	0.287	0.007 *
	CG	12.10	1.14	12.30	10.40	14.30	9.39	11.90	1.20	11.80	9.80	14.90	10.15	0.051	0.287
	*p* int	0.719						0.812							
	ICC	0.17													
PDW [%]	G1	15.70	0.28	15.70	15.10	16.20	1.75	15.70	0.27	15.70	15.00	16.20	1.71	0.014	0.600
	G2	15.80	0.19	15.80	15.50	16.10	1.19	15.80	0.30	15.80	14.90	16.20	1.91	0.009	0.651
	CG	15.70	0.27	15.70	15.10	16.40	1.75	15.70	0.27	15.70	15.20	16.4	1.74	0.014	0.567
	*p* int	0.253						0.296							
	ICC	0.70													
PCT [%]	G1	0.29	0.09	0.28	0.16	0.62	31.20	0.28	0.07	0.28	0.12	0.41	23.33	0.007	0.751
	G2	0.28	0.06	0.29	0.19	0.45	21.97	0.28	0.07	0.29	0.17	0.42	23.80	0.000	0.765
	CG	0.29	0.06	0.30	0.18	0.38	19.00	0.29	0.06	0.29	0.14	0.39	20.09	0.000	0.750
	*p* int	0.910						0.958							
	ICC	−0.33													

$\bar{X}$ —mean, SD—standard deviation, Me—median, Min—minimum, Max—maximum, CV—coefficient of variation, ES—effect size, ICC—intraclass correlation coefficient, *p*—* statistically significant value (*p* < 0.05), WBC—White Blood Cells, PLT—Platelets, MPV—Mean Platelet Volume, PDW—Platelet Distribution Width, PCT—Plateletcrit.

**Table 5:** Values of reticulocytes parameters in the examined (G1, *n* = 23; G2, *n* = 23), and control (CG, *n* = 23) groups before and after vibrotherapy series, considering interactions between the groups (*p* int) and changes over time (*p* pre-post).

Variable	Group	Pre						Post						ES	*p* Value
		$\bar{X}$	SD	Me	Min	Max	CV	$\bar{X}$	SD	Me	Min	Max	CV		Pre-Post
RETC [%]	G1	15.90	3.75	15.70	8.80	24.40	23.56	15.70	3.62	15.50	9.20	25.00	23.08	0.104	0.650
	G2	16.50	2.78	15.60	11.90	20.80	16.85	16.20	2.99	15.90	11.30	21.30	18.44	0.158	0.413
	CG	17.50	4.66	16.00	9.00	27.70	26.68	17.00	3.68	16.70	9.70	24.10	21.62	0.202	0.418
	*p* int	0.390						0.425							
	ICC	0.60													
HFR [%]	G1	2.40	1.78	1.60	0.30	6.60	75.30	1.40	1.42	1.10	0.00	6.50	101.11	0.376	<0.001 *
	G2	2.20	2.33	1.60	0.70	12.00	104.31	1.10	0.57	1.00	0.00	2.30	52.22	0.247	0.013 *
	CG	2.50	2.45	1.70	0.40	11.00	97.17	1.90	2.19	1.00	0.10	8.70	116.43	0.205	0.026 *
	*p* int	0.904						0.232							
	ICC	0.36													
LRF [%]	G1	87.90	4.28	88.40	80.40	96.30	4.86	90.40	3.86	89.70	80.70	97.70	4.27	0.755	<0.001 *
	G2	87.80	3.73	88.20	74.30	92.80	4.25	90.60	2.52	90.40	85.40	94.20	2.78	0.788	<0.001 *
	CG	87.70	5.40	88.00	74.50	94.50	5.40	89.60	4.82	91.20	75.90	94.10	5.38	0.671	<0.001 *
	*p* int	0.983						0.671							
	ICC	−0.11													
MFR [%]	G1	9.70	2.88	10.00	3.40	14.60	29.73	8.20	2.74	8.40	2.30	12.80	33.25	0.472	<0.001 *
	G2	9.90	1.93	10.20	6.30	13.70	19.39	8.30	2.05	8.10	4.90	12.40	24.68	0.439	<0.001 *
	CG	9.70	2.61	10.30	5.10	14.10	26.83	8.50	2.88	7.00	5.00	15.40	34.01	0.351	0.002 *
	*p* int	0.942						0.951							
	ICC	−0.40													
IRF [%]	G1	12.10	4.28	11.60	3.70	19.60	35.47	9.60	3.86	10.30	2.30	19.30	40.00	0.749	<0.001 *
	G2	12.20	3.73	11.80	7.20	25.70	30.64	9.40	2.52	9.60	5.80	14.60	26.75	0.788	<0.001 *
	CG	12.30	4.74	12.00	5.50	25.50	38.56	10.30	4.84	7.90	5.90	24.10	46.77	0.678	<0.001 *
	*p* int	0.983						0.695							
	ICC	−0.13													

$\bar{X}$ —mean, SD—standard deviation, Me—median, Min—minimum, Max—maximum, CV—coefficient of variation, ES—effect size, ICC—intraclass correlation coefficient, *p*—* statistically significant value (*p* < 0.05), RETC—number of reticulocytes, HFR—high-fluorescence reticulocytes, LFR—low-fluorescence reticulocytes, MFR—medium-fluorescence reticulocytes, IRF—immature-reticulocyte fraction.

**Table 6:** Values of indexes of erythrocyte aggregation and fibrinogen concentration in the examined (G1, *n* = 23; G2, *n* = 23), and control (CG, *n* = 23) groups before and after vibrotherapy series, considering interactions between the groups (*p* int) and changes over time (*p* pre-post).

Variable	Group	Pre						Post						ES	*p* Value
		$\bar{X}$	SD	Me	Min	Max	CV	$\bar{X}$	SD	Me	Min	Max	CV		Pre-Post
AMP [au]	G1	17.87	5.73	17.80	10.49	29.18	32.05	20.59	2.83	20.57	14.25	27.64	13.77	0.160	0.053
	G2	19.60	4.72	20.81	11.10	26.39	24.08	21.34	4.45	22.80	10.78	27.01	20.86	0.053	0.280
	CG	18.57	4.27	19.05	8.97	24.74	22.98	18.75	4.30	19.46	27.01	26.20	22.94	0.001	0.873
	*p* int	0.494						0.079							
	ICC	0.68													
T ½ [s]	G1	2.19	1.22	1.97	0.03	5.48	55.87	2.11	0.82	2.15	0.09	3.92	39.11	0.004	0.779
	G2	1.86	0.98	1.89	0.00	3.54	52.52	2.34	0.86	2.12	1.03	4.07	36.81	0.197	0.030 *
	CG	2.32	0.74	2.05	1.09	3.76	32.10	2.11	0.64	2.02	1.06	3.83	30.44	0.074	0.200
	*p* int	0.276						0.513							
	ICC	0.75													
AI [%]	G1	63.60	10.70	64.800	42.70	89.30	16.79	62.60	11.48	60.30	48.50	77.70	11.48	0.008	0.679
	G2	66.40	11.70	64.20	51.60	99.10	17.58	61.70	11.89	62.90	48.0	74.50	11.89	0.151	0.009 *
	CG	61.70	6.60	64.20	50.60	75.10	10.77	62.00	10.32	62.90	48.50	74.50	10.32	0.003	0.796
	*p* int	0.281						0.908							
	ICC	0.68													
Fib [g/L]	G1	3.43	0.59	3.57	2.15	4.33	17.23	3.27	0.52	3.34	2.40	4.83	15.83	0.070	0.212
	G2	3.47	0.75	3.42	2.62	5.83	21.75	3.22	0.50	3.17	2.41	4.34	15.58	0.194	0.031 *
	CG	3.34	0.47	3.34	2.50	4.16	14.13	3.11	0.35	3.28	2.58	4.10	11.24	0.290	0.007 *
	*p* int	0.782						0.512							
	ICC	0.27													

$\bar{X}$ —mean, SD—standard deviation, Me—median, Min—minimum, Max—maximum, CV—coefficient of variation, ES—effect size, ICC—intraclass correlation coefficient, *p*—* statistically significant value (*p* < 0.05), AMP—amplitude of aggregation, T ½—half time kinetics of aggregation, AI—aggregation index, Fib—fibrinogen.

**Table 7:** Values of the protein profile parameters in the examined (G1, *n* = 23; G2, *n* = 23), and control (CG, *n* = 23) groups before and after vibrotherapy series, considering interactions between the groups (*p* int) and changes over time (*p* pre-post).

Variable	Group	Pre						Post						ES	*p* Value
		$\bar{X}$	SD	Me	Min	Max	CV	$\bar{X}$	SD	Me	Min	Max	CV		Pre-Post
Total proteins [g/L]	G1	72.97	3.48	72.30	67.50	81.60	4.77	72.46	3.41	72.40	65.10	79.60	4.70	0.025	0.465
	G2	73.02	3.17	73.40	65.80	80.50	4.34	71.10	3.07	70.50	65.20	77.50	4.32	0.265	0.010 *
	CG	75.23	3.63	75.00	67.60	83.60	4.82	73.13	3.24	72.60	66.80	78.80	4.43	0.444	<0.001 *
	*p* int	0.045 *						0.106							
	ICC	0.81													
Albumins [g/L]	G1	42.74	2.66	42.70	38.20	48.40	6.23	42.61	2.73	42.30	36.80	47.90	6.41	0.002	0.819
	G2	43.00	1.23	43.00	40.70	45.20	2.86	41.93	2.61	42.20	33.60	46.50	6.23	0.144	0.068
	CG	43.34	2.45	43.30	38.00	48.50	5.66	42.87	2.43	42.20	39.80	47.60	5.68	0.025	0.458
	*p* int	0.650						0.455							
	ICC	0.34													
α-1-globulins [g/L]	G1	1.61	0.18	1.60	1.30	2.00	11.23	1.59	0.25	1.60	1.00	2.20	16.04	0.005	0.725
	G2	1.57	0.25	1.60	1.20	2.10	16.17	1.63	0.49	1.50	1.00	3.40	30.17	0.025	0.461
	CG	1.54	0.28	1.50	1.00	2.30	18.45	1.51	0.26	1.40	1.20	2.20	17.18	0.012	0.615
	*p* int	0.628						0.505							
	ICC	0.38													
α-2-globulins [g/L]	G1	8.24	0.82	8.10	6.50	9.30	10.00	8.09	1.13	7.90	6.10	10.90	14.03	0.014	0.578
	G2	8.07	0.88	8.00	5.80	9.50	10.92	7.89	1.21	7.80	5.10	11.40	15.35	0.060	0.247
	CG	8.11	0.75	8.10	6.20	9.80	9.30	7.71	0.91	7.90	5.90	9.20	11.77	0.096	0.141
	*p* int	0.764						0.497							
	ICC	0.35													
β-1-globulins [g/L]	G1	6.63	1.04	6.08	5.33	9.39	15.62	6.57	1.01	6.52	4.96	8.77	15.33	0.004	0.767
	G2	6.60	0.99	6.43	4.96	8.66	15.06	6.50	0.80	6.40	4.89	7.79	12.25	0.011	0.622
	CG	6.84	0.93	6.61	5.59	9.11	13.59	6.89	0.96	7.12	5.27	8.83	14.00	0.004	0.768
	*p* int	0.677						0.326							
	ICC	0.52													
β-2-globulins [g/L]	G1	4.68	0.96	4.50	3.17	6.43	20.59	4.58	1.22	4.47	2.69	7.23	26.57	0.019	0.525
	G2	4.65	0.96	4.39	3.47	6.76	20.77	4.39	1.02	4.26	2.93	6.91	23.16	0.081	0.178
	CG	5.16	0.87	5.11	3.77	6.98	16.92	4.72 *	0.97	5.21	3.18	6.54	20.47	0.185	0.036 *
	*p* int	0.119						0.580							
	ICC	0.65													
γ-globulins [g/L]	G1	9.09	1.65	8.50	6.50	12.60	18.27	9.02	1.55	9.00	5.00	11.90	17.19	0.003	0.796
	G2	9.14	2.02	9.10	5.90	13.10	22.06	8.78	1.99	8.30	5.70	13.10	22.63	0.130	0.083
	CG	10.09	3.16	9.40	5.40	20.20	31.36	9.47	2.41	9.00	5.10	16.10	25.49	0.174	0.042 *
	*p* int	0.279						0.510							
	ICC	0.61													

$\bar{X}$ —mean, SD—standard deviation, Me—median, Min—minimum, Max—maximum, CV—coefficient of variation, ES—effect size, ICC—intraclass correlation coefficient, *p*—* statistically significant value (*p* < 0.05).

## Data Availability

Data are available on request from the corresponding author.
